# Muscle contraction velocity, strength and power output changes following different degrees of hypohydration in competitive olympic combat sports

**DOI:** 10.1186/s12970-016-0121-3

**Published:** 2016-03-08

**Authors:** J. G. Pallarés, A. Martínez-Abellán, J. M. López-Gullón, R. Morán-Navarro, E. De la Cruz-Sánchez, R. Mora-Rodríguez

**Affiliations:** Human Performance and Sports Science Laboratory, University of Murcia, Murcia, Spain; Exercise Physiology Laboratory, University of Castilla-La Mancha, Toledo, Spain

**Keywords:** Hydration, Urine osmolality, Bench press, Taekwondo, Olympic wrestling and boxing

## Abstract

**Background:**

It is habitual for combat sports athletes to lose weight rapidly to get into a lower weight class. Fluid restriction, dehydration by sweating (sauna or exercise) and the use of diuretics are among the most recurrent means of weight cutting. Although it is difficult to dissuade athletes from this practice due to the possible negative effect of severe dehydration on their health, athletes may be receptive to avoid weight cutting if there is evidence that it could affect their muscle performance. Therefore, the purpose of the present study was to investigate if hypohydration, to reach a weight category, affects neuromuscular performance and combat sports competition results.

**Methods:**

We tested 163 (124 men and 39 woman) combat sports athletes during the 2013 senior Spanish National Championships. Body mass and urine osmolality (U_OSM_) were measured at the official weigh-in (PRE) and 13–18 h later, right before competing (POST). Athletes were divided according to their U_SOM_ at PRE in euhydrated (EUH; U_OSM_ 250–700 mOsm · kgH_2_O^−1^), hypohydrated (HYP; U_OSM_ 701–1080 mOsm · kgH_2_O^−1^) and severely hypohydrated (S-HYP; U_OSM_ 1081–1500 mOsm · kgH_2_O^−1^). Athletes’ muscle strength, power output and contraction velocity were measured in upper (bench press and grip) and lower body (countermovement jump - CMJ) muscle actions at PRE and POST time-points.

**Results:**

At weigh-in 84 % of the participants were hypohydrated. Before competition (POST) U_OSM_ in S-HYP and HYP decreased but did not reach euhydration levels. However, this partial rehydration increased bench press contraction velocity (2.8-7.3 %; *p* < 0.05) and CMJ power (2.8 %; *p* < 0.05) in S-HYP. Sixty-three percent of the participants competed with a body mass above their previous day’s weight category and 70 of them (69 % of that sample) obtained a medal.

**Conclusions:**

Hypohydration is highly prevalent among combat sports athletes at weigh-in and not fully reversed in the 13–18 h from weigh-in to competition. Nonetheless, partial rehydration recovers upper and lower body neuromuscular performance in the severely hypohydrated participants. Our data suggest that the advantage of competing in a lower weight category could compensate the declines in neuromuscular performance at the onset of competition, since 69 % of medal winners underwent marked hypohydration.

## Background

Olympic weight-class combat sports (i.e., wrestling, boxing, judo and taekwondo) match their athletes before competition, separating them by sex and weight class. Weight class divisions have the purpose of making competition fair by matching opponents of similar muscle mass and strength and by doing so, reducing the risk of injury [1, 2]. However, the timing between weigh-in and competition can turn this noble purpose into a perverse rule. In Olympic weight-class sports the official weigh-in is typically held 6–24 h before competition for both the amateur and professional fighters. This allows most athletes to use aggressive weight-cut practices to lose weight and enter competition in a lower weight class, followed by fast weight regain during the hours before competition.

In 1997 dehydration ranging from 7–10 % contributed to the death of three collegiate level wrestlers in the USA. Those episodes persuaded authorities to advance the weigh-in period to 1–2 h before competition in high school and collegiate wrestling. Unfortunately, this rule has not spread into the European or international federations of any combat sport and, as a consequence, weight-cutting practices prevail [3]. The American College of Sports Medicine (ACSM) has, for long time, been suggesting that athletes should not lower their body mass below the weight at which body fat levels are lower than 5 % [4]. Thus, minimal combat weight has habitually been established based on body fat estimations (i.e., skinfolds or bioimpedance [5]), while educational programs have been administered [6] to prevent rapid weight loss practices inducing severe dehydration in combat athletes. Nevertheless, the success of these educational programs is tenuous [7] since approximately one third of these athletes compete below their calculated minimal weight and are still very successful [8].

The reluctance of the combat sports ruling authorities to impede these weight-cut practices despite the accumulation of scientific literature showing the negative effects of hypohydration on performance and health seems peculiar. However, it is understandable that athletes and their coaches do not take into account the negative effects of hypohydration on their physical performance, compared to the advantage of competing in a lower weight category. To our knowledge, one study stands alone addressing whether competition results are negatively correlated with body mass gained between weigh-in and competition (an index of initial dehydration). The results reported in that study do not discourage athletes from undergoing weight cutting practices [8]. This is, 60 % of the wrestlers below their minimal competition weight ranked among the first 4 in each category, while only 33 % of the wrestlers in their natural combat body mass were winners during the competition.

Losses of fat mass require weeks of dieting, and losses of the carbohydrate stored in the body can only account for ~0.5 kg of body mass loss. Thus, most of the weight loss achieved by these combat sport athletes during weight-cut is due to loss of body water. Extreme reductions in fluid and food intake, sauna exposure, diuretic pills use [7] and exercise using rubberized sweat suits are common means used by combat sport athletes to reduce body mass during the days prior to weigh-in [2]. Hypohydration negatively impacts on the capacity of the body to thermoregulate, resulting in increased core temperature during exercise [9]. In turn, hyperthermia has been repeatedly shown to result in premature fatigue during intense aerobic exercise [10, 11]. In addition, hypohydration and the resulting hyperthermia, induces cardiovascular drift, which is associated with performance impairments such as declines in cycling peak power [12], anaerobic power and maximal aerobic capacity [13].

Although success in Olympic combat sports is multifactorial, recent studies have shown that muscle strength and power are key factors affecting performance in these sports [1, 14]. However, the effects of rapid dehydration and rehydration on neuromuscular performance (i.e., muscle strength and power) have not been sufficiently explored. Weight loss by dehydration has been shown to affect boxing and wrestling performance [13, 15]. However, if the weight loss is quickly recovered, the effects on performance are not evident [16]. Maximal isometric muscle strength is found to be either reduced [17] or unchanged [18] after rapid weight loss. Montain and co-workers found that 4 % dehydration reduced muscle endurance, but not isokinetic knee extension strength, while neither pH or Pi were affected by hypohydration [19]. Nevertheless, recent studies argue that hypohydration may impair isometric-eccentric strength, and particularly the rate of force development [20, 21]. Therefore, the purpose of this study was to determine whether the hypohydration which competitive Olympic combat sport athletes undergo to get into a lower weight-class category, reduces their neuromuscular performance. In addition, we investigated whether the magnitude of body mass gained between the weigh-in and the beginning of competition (an index of initial dehydration) is related to the performance results in a real competition event.

## Methods

### Subjects

One hundred and twenty-four male (age 22.4 ± 4.3 years, body mass 75.1 ± 14.7 kg, height 177.1 ± 7.1 cm) and thirty nine female (age 22.7 ± 4.4 years, body mass 56.9 ± 8.8 kg, height 164.7 ± 7.0 cm) high performance athletes of three different Olympic combat sports volunteered to participate in this study: wrestling (*n* = 76), taekwondo (*n* = 62) and boxing (*n* = 25). This sample can be considered representative of the population studied, not only for its size in absolute terms, but because these 163 participants represented 61.7 % of all athletes competing at the 2013 Spanish National Championship in these three Olympic combat sports. All participants had at least 4 years of training and competition experience. Athletes and coaches were informed in detail about the experimental procedures and the possible risks and benefits of the project. The study complied with the Declaration of Helsinki and was approved by the Bioethics Commission of the University of Murcia. Written informed consent was obtained from athletes prior to participation. The four medal winners of each weight category (1^st^, 2^nd^ and the two 3^rd^ classified) in the Spanish National Championship were grouped as the Elite group. The remaining participants were assigned to the non-Elite group [1, 14].

### Experimental approach to the problem

Athletes’ body mass, hydration status and neuromuscular performance were evaluated on two separate occasions: *i*) between 60 and 5 min before the official weigh-in of their respective National Championship (PRE) and, *ii*) between 60 and 5 min before the beginning of the first combat bout (POST). PRE trials were conducted between 16:00 and 19:00 h, and POST trial between 8:00 and 10:00 h the following day. No instructions were given to athletes or their coaches about weight control management. Eight subjects were excluded from the study for ingesting vitamins, nutritional supplements or prescription drugs prone to alter urine composition [22]. Three women were excluded because they were in the proliferative phase of their menstrual cycle which could alter urine composition. In addition, 20 subjects were not allowed to participate in the study due to lack of familiarization with the weight lifting procedures.

At arrival to the testing facilities, a 10 ml mid flow urine sample was obtained from each athlete. After the tube with the urine sample was handed over and codified, subjects’ body mass was determined and fat-free mass percentage estimated using a calibrated scale (Tanita BC-418, Tanita Corp., Tokyo, Japan). Urine specimens were immediately analyzed for urine osmolality (U_OSM_) by the same experienced investigator. After a standardized warm-up that consisted of 5 min of joint mobilization exercises, the subjects entered the laboratory to start the neuromuscular test battery assessments under a paced schedule. These tests consisted of *i*) the measurement of bar displacement velocity against 3 to 5 incremental loads in the bench press exercise, *ii*) maximal isometric grip strength test, and *iii*) countermovement jump height test. Only participants who reported being familiar with these resistance training exercises were included in the study (final sample 163 combat sport athletes). Neuromuscular testing was completed for all participants in the three National Championships and two time points (PRE and POST) at the same human performance laboratory close to the official weigh-in and competition facilities.

### Procedures

#### Isoinertial strength assessment

After a warm-up for bench press exercise (i.e., 2 sets of 10 repetitions against 20 kg and 35 kg) all participants performed a graded submaximal loading test in a Smith machine (Multipower Fitness Line, Peroga, Spain) with a linear encoder and its associated software (T-Force System, Ergotech, Murcia, Spain, 0.25 % accuracy) attached to the bar by a light retractable metal cable. The initial load was set at 20 kg for all participants and was gradually increased by 20 kg for men and 10 kg for women until mean propulsive velocity was between 0.60 m · s^−1^ and 0.50 m · s^−1^ (~70–80 % 1RM; [23]). To attain that propulsive velocity each participant lifted between 3 to 5 increasing loads. The same warm up and loads performed during the PRE trial (i.e., before the official weigh-in) were replicated in the POST trial (i.e., before the beginning of the tournament).

Mean propulsive velocity (MPV) was calculated as the average velocity measured only during the propulsive phase, defined as the portion of the concentric action during which the bar acceleration is greater than acceleration due to gravity [23]. In each trial (PRE and POST), three repetitions were executed for light (MPV between 1.40 and 1.00 m · s^−1^), two for medium (MPV between 0.99 and 0.75 m · s^−1^), and only one for the heaviest (MPV between 0.74 and 0.50 m · s^−1^) loads interspersed with 5-min of passive rest.

Individual range of movement was carefully replicated in each trial with the help of two telescopic bar holders with a precision of ± 1.0 cm. The bar holders were positioned to allow the bar to descend to 1 cm off each subject’s chest. Subjects were instructed to perform the eccentric phase in a slow and controlled manner, remain paused for 2 s at the bar holders, momentarily releasing the weight, and thereafter to perform a purely concentric action, pushing at the maximal possible velocity. The momentary pause imposed between the eccentric and concentric actions was designed to minimize the contribution of the stretch-shortening cycle (i.e., rebound effect) and allow for a more reliable and consistent measurement [23].

Given the close relationship between the bar mean propulsive velocity (i.e., MPV) attained during the concentric phase and the load (% 1RM; r > 0.995 [24]) it was not necessary to reach the one repetition maximum load (1RM) to quantify the effects that the 13–18 h that separated the PRE and the POST time points had on neuromuscular performance. We analyzed the MPV difference between the PRE and POST trials against the heaviest load lifted during the incremental loading test (MPV between 0.60 and 0.50 m · s^−1^). As recently reported, the MPV at this load represents the most valid and reliable test to predict maximum strength through bar displacement velocity [23, 24]. In addition, this procedure avoids the physical and mental stress associated with 1RM assessment, it drastically reduces testing time and injury risk.

#### Jumping test (CMJ)

Participants completed three repetitions of a countermovement vertical jump (CMJ). For this test, participants squatted down into a self-selected depth prior to explosively performing the concentric action. Participants were instructed to keep their hands on their hips at all times and to maintain the same position at take-off and landing. Flight times were measured using an infrared jump system (Optojump, Microgate, Italy). The recorded height for this test was the average of three trials. Absolute mechanical power during CMJ was calculated with the following formula: CMJ_P_ = BM g (2 g h)^1/2^ in which “BM” is body mass in kg, “g” the acceleration of gravity in m s^−2^, and “h” the jumping height in meters. The detailed testing procedures, validity, and reliability (i.e., test–retest ICC and CV were 0.94 and 3.3 %, respectively) have recently been established elsewhere [1].

#### Maximal hand grip strength tests

Each subject’s grip strength was measured for dominant and non-dominant hands with a Baseline Hydraulic Dynamometer (Country Technology Inc; Gays Mills, USA). Participants sat with 0° of shoulder flexion and 90° of elbow flexion and the forearm in neutral position. The average result of two repetitions with each arm was recorded.

#### Urine osmolality

U_OSM_ is the measure of the total urine solute content. As has been repeatedly reported, this procedure is considered the gold standard non-invasive measure to determine the athletes’ hydration status [3, 25]. Upon collection, athlete’s urine specimens (10 mL) were immediately analyzed in duplicate by freezing point depression osmometry (Model 3250, Advanced Instruments, USA).

### Statistical analysis

Standard statistical methods were used for the calculation of means, standard deviation (SD), standard error of the means (SEM) and effect size (ES). Subjects were stratified in three groups according to their hydration status based on U_OSM_ values [26] at PRE data collection. As recently described [3] three intervals of equal amplitude were established according to the following cutoff values: from 250 to 700 mOsm · kg H_2_O^−1^ (euhydrated - EUH; *n* = 26), from 701 to 1.080 mOsm · kg H_2_O^−1^ (hypohydrated - HYP; *n* = 69) and from 1.081 to 1.500 mOsm · kg H_2_O^−1^ (severely hypohydrated - S-HYP; *n* = 68). The Shapiro-Wilk test was used to assess normal distribution of data. A two-way (hydration level x time) ANOVA was used to detect differences in U_OSM_ values. One-way ANOVA was run for comparison of change scores (PRE vs. POST) in body composition and neuromuscular performance variables. The Greenhouse-Geisser adjustment for sphericity was calculated. After a significant F test, differences among means were identified using pairwise comparisons with Bonferroni’s adjustment. Differences in the change scores between the elite and non-elite groups were analysed using Student’s *t* test. Additionally, a chi-square test for association was conducted between competitive levels and hydration status, with all expected cell frequencies greater than five. The *p* < 0.05 criterion was used for establishing statistical significance.

## Results

### Urine osmolality and body mass

**Fig. 1:**
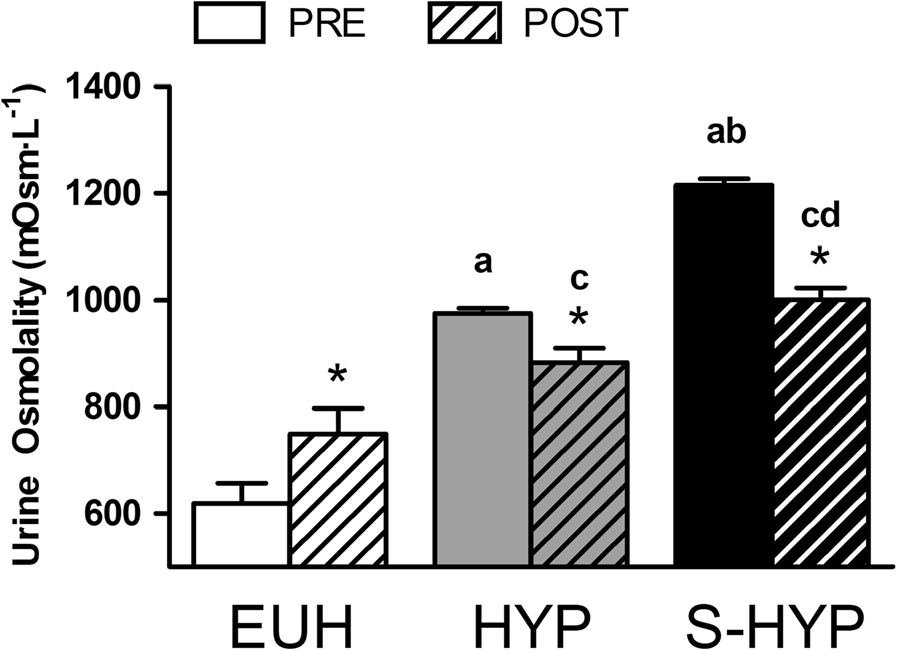
Changes in urine osmolality between PRE and POST time points for each group (EUH, HYP and S-HYP). Data are presented as mean ± SEM. *Significant difference respective to the PRE time point. ^a^ significant difference when compared to EUH at PRE; ^b^ when compared to HYP at PRE; ^c^ when compared to EUH at POST; ^d^ when compared to HYP at POST

When the PRE urine osmolality results of the three groups were compared, S-HYP 1215 ± 95 mOsm · L^−1^; SEM = 12 mOsm · L^−1^) showed significantly higher values than HYP (975 ± 79 mOsm · L^−1^ SEM = 9 mOsm · L^−1^; 24.6 % higher; ES = 2.74; *p* < 0.001) and EUH (618 ± 187 mOsm · L^−1^; SEM = 37 mOsm · L^−1^; 96.4 % higher; ES = 4.22; *p* < 0.001). Significantly higher U_OSM_ values were detected in the HYP compared to EUH in PRE (57.7 % higher; ES = 2.67; *p* < 0.001). Similarly, POST U_OSM_ in S-HYP (1000 ± 185 mOsm · L^−1^; SEM = 22 mOsm · L^−1^) was significantly higher than HYP (883 ± 224 mOsm · L^−1^; SEM = 26 mOsm · L^−1^; 13.3 % higher; ES = 0.57; *p* < 0.02) and EUH (749 ± 238 mOsm · L^−1^; SEM = 48 mOsm · L^−1^; 33.5 % higher; ES = 1.18; *p* < 0.001). Also, significant higher values were detected in the HYP compared to EUH at POST (17.8 % higher; ES = 0.58; *p* < 0.001; Fig. 1).

U_OSM_ changed between PRE and POST time points in all groups. The euhydrated group (EUH) significantly increased its osmolality in the 13–18 h that separated the official weigh-in and the beginning of the tournament (21.2 %; ES = 0.61; *p* < 0.01). By contrast, the hypohydrated (HYP) and severely hypohydrated (S-HYP) groups significantly decreased their urine osmolality (i.e., rehydration) in the same period of time (HYP = −9.5 %, ES = 0.61; *p* < 0.01; S-HYP = −17.7 %, ES = 1.53; *p* < 0.01; Fig. 1).

**Fig 2:**
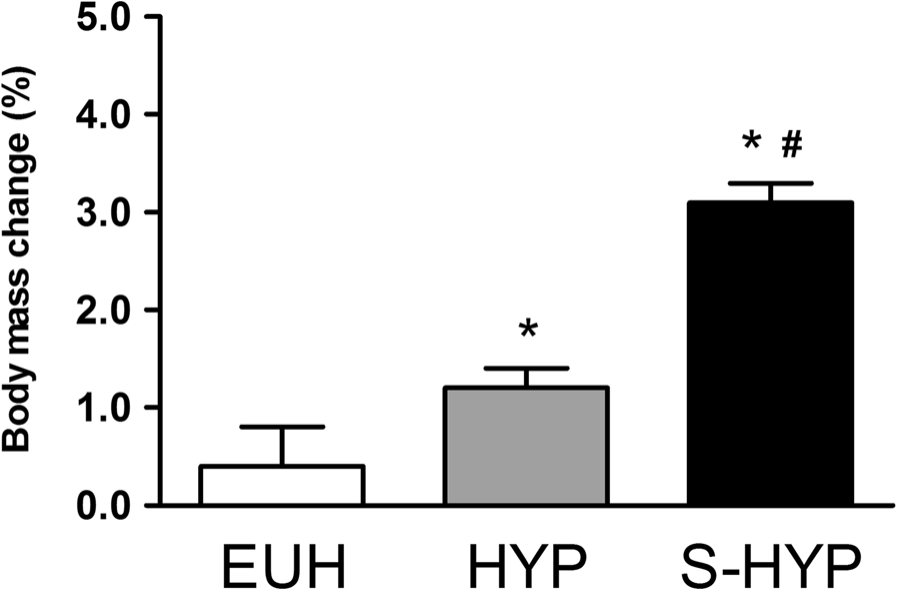
Relative changes in body mass for each group (EUH, HYP and S-HYP). Data are presented as mean ± SEM. Significant differences ^a^ when compared to EUH; ^b^ when compared to HYP

Body mass difference between PRE and POST time points (i.e., another index of rehydration) did not change for the euhydrated group (i.e., EUH). However, the hypohydrated group HYP recovered 1.2 % of body mass and the group severely hypohydrated (i.e., S-HYP) recovered 3.1 % of body mass. The recovery in the S-HYP group was higher than the recovery in the rest of the groups (ES = 0.49 – 1.41; *p* < 0.05; Fig. 2).

**Fig. 3:**
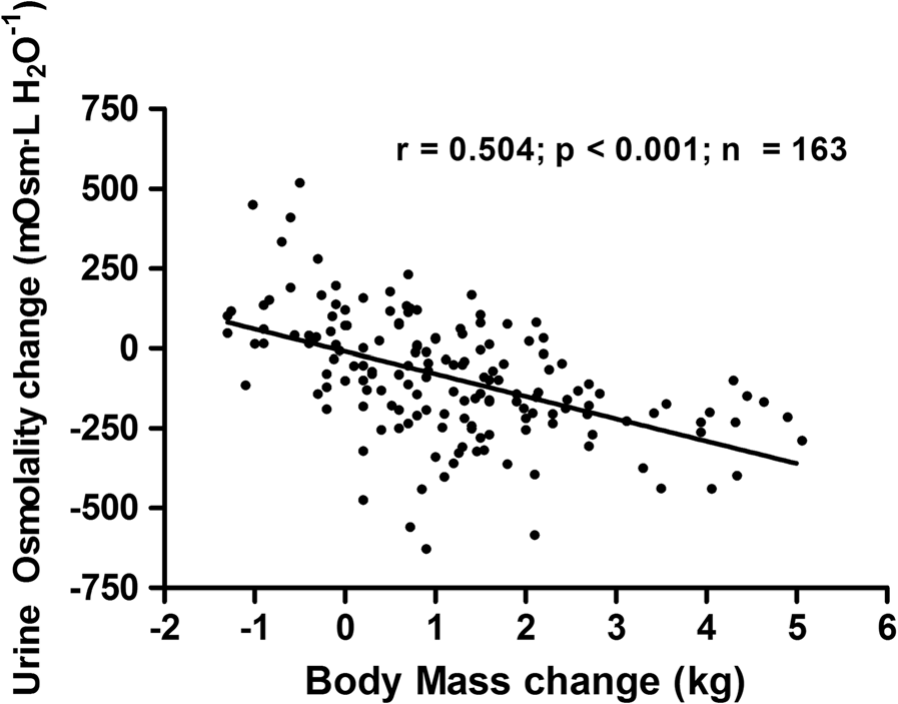
Correlation between U_OSM_ changes and body mass changes between PRE and POST time points in the whole sample

A strong linear negative correlation was detected between body mass changes and U_OSM_ changes between the PRE and POST time points in the whole sample (*r* = 0.504; *p* < 0.001; *n* = 163; Fig. 3).

### Neuromuscular assessments

**Fig. 4:**
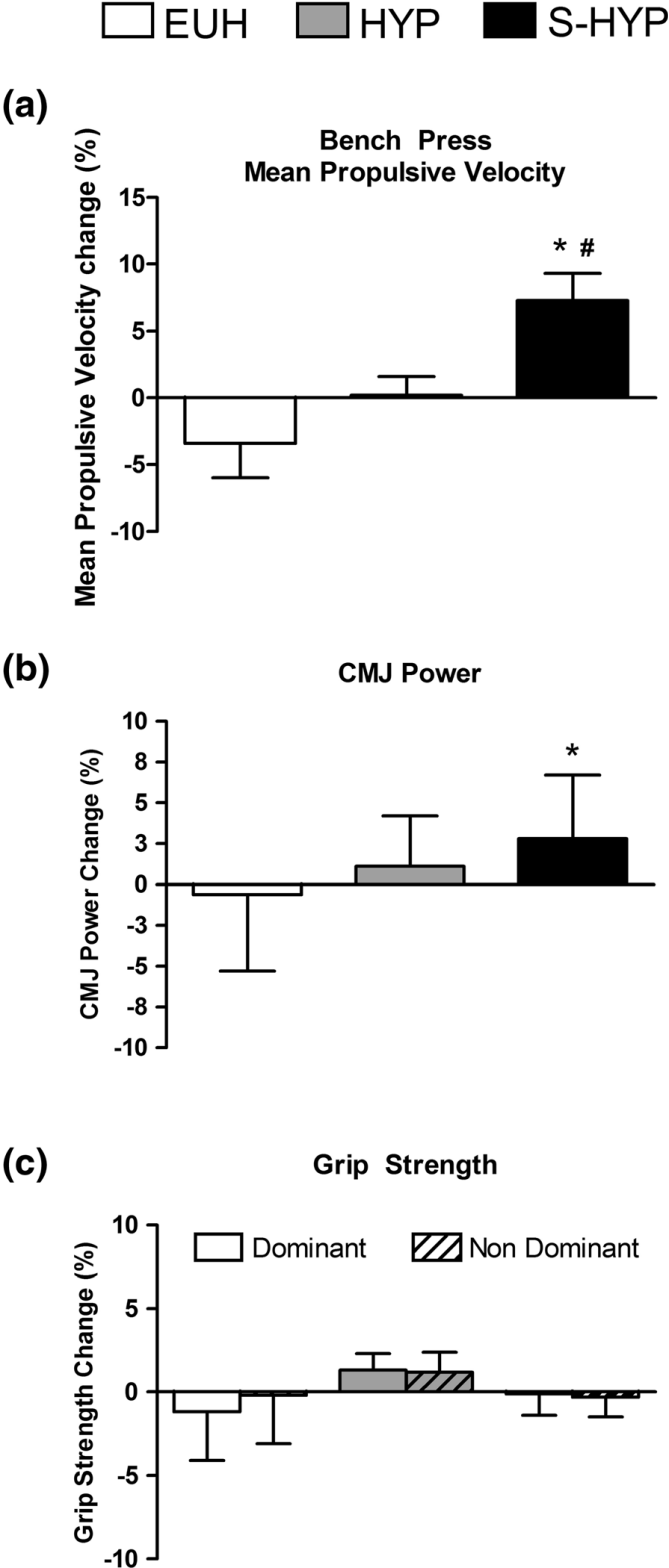
Relative changes in (**a**), bench press mean propulsive velocity, (**b**) countermovement jump power and (**c**) grip strength (between PRE and POST time points for each group (EUH, HYP and S-HYP). Data are presented as mean ± SEM. ^a^ Significantly different compared to EUH. ^b^ Significantly different compared to HYP

The increases in MPV in the severely hypohydrated group (i.e., S-HYP) was higher than in the EUH and HYP groups (7.3 ± 2.6 % compared to −3.4 ± 2.6 % for EUH and 0.2 ± 1.4 % for HYP; *p* < 0,001; Fig. 4a). Likewise, significantly higher CMJ power increases were detected in the S-HYP group (2.8 ± 3.9 %) compared to EUH (−0.6 ± 4.7 %; ES = 0.79; *p* < 0.001) and close to significance when compared to HYP (1.1 ± 3.1 %; ES = 0.49; *p* = 0.08; Fig. 4b). No significant differences were detected in the relative changes of maximum grip strength for dominant or non-dominant hands, neither between PRE and POST values, nor between groups (EUH, HYP and S-HYP) at any time point (Fig. 4c).

### Results by competitive level

**Fig. 5:**
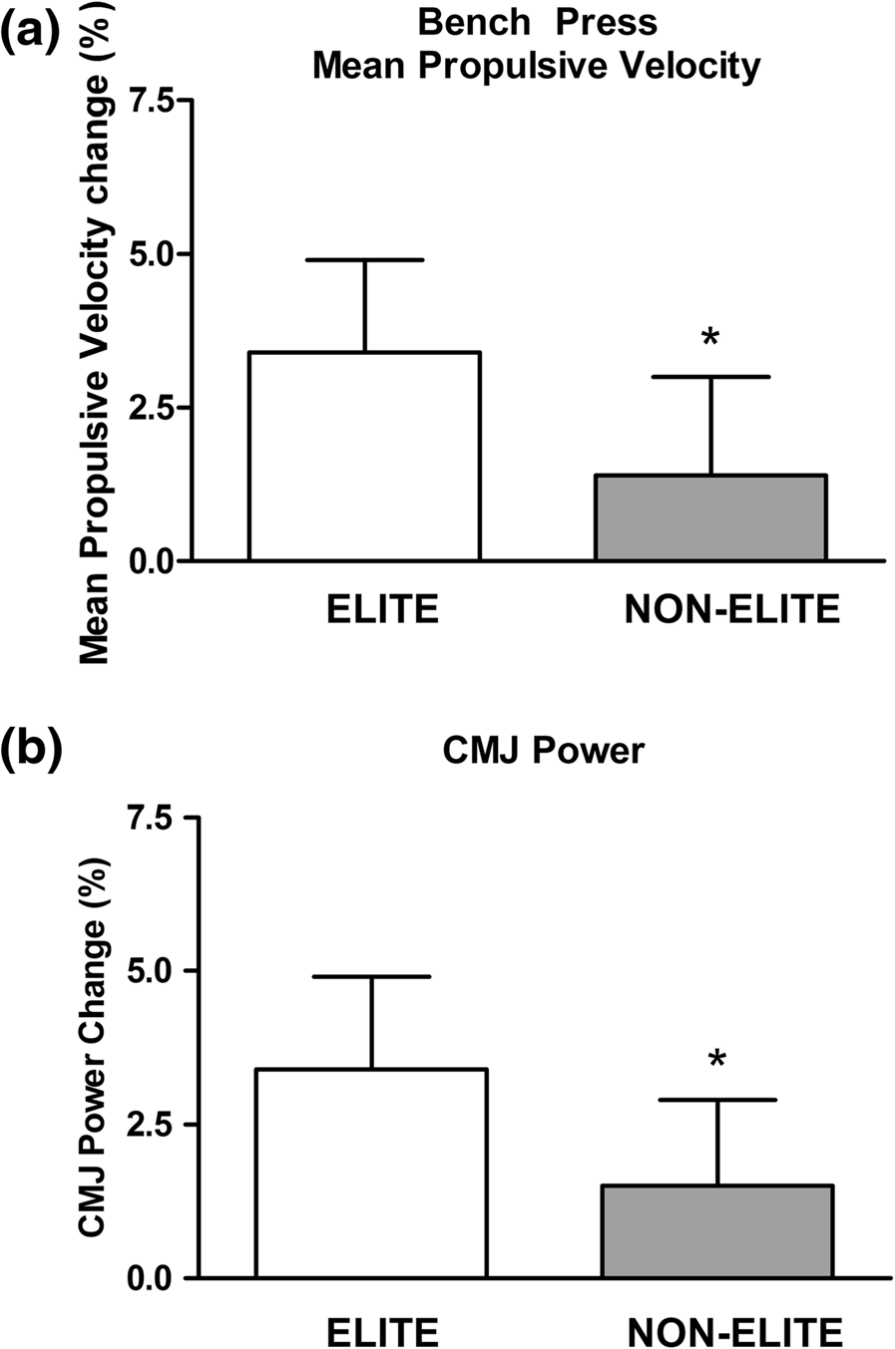
Relative changes in (**a**) bench press mean propulsive velocity and (**b**) countermovement jump power between PRE and POST testing for Elite and non-Elite groups. Data are presented as mean ± SEM. *Significantly different when compared to Elite

When PRE hydration status of all medals winners at their respective National Championships (Elite) were compared to the remaining competitors (i.e., non-Elite), Elite showed higher mean urine osmolality (Elite = 1034.3 ± 28.6 mOsm · kg H_2_O^−1^; non-Elite = 955.8 ± 22.4 mOsm · kg H_2_O^−1^; *p* < 0.05). Chi-squared test showed a significantly higher proportion of severe dehydration (U_OSM_ > 1080 mOsm · kg H_2_O^−1^) in the Elite group (46.8 % vs. 26.2 %; *χ*2(2) = 7.519, *p* = 0.023). However, no significant differences were detected between competitive levels before the beginning of the tournament (i.e., POST; Elite = 906.8 ± 28.1 mOsm · kg H_2_O^−1^; non-Elite = 888.0 ± 22.9 mOsm · kg H_2_O^−1^). Larger MPV changes were detected between PRE and POST in the Elite group compared to the non-Elite (3.4 ± 2.6 % vs. 1.4 ± 2.5; *p* < 0.05; Fig. 5a). Also, significant higher increases in CMJ power were detected in the Elite group compared to the non-Elite athletes (3.4 ± 4.7 % vs. 1.5 ± 4.1; *p* < 0.05; Fig. 5b).

Out of the 163 participants in this study, 102 (i.e., 63 %) started the competition with a body mass above the weight category they entered at weigh-in (i.e., 13–18 h before competition). Thus, those 102 participants are suspected of having undergone dehydration to cut weight. Of these 102 athletes, 70 of them obtained a medal (i.e., Elite) in the championship. This is, 69 % of all the hypohydrated athletes at weigh-in were successful at competition. Conversely, only an 11 % of the athletes obtaining a medal did not enter a lower weight category at weigh-in, and thus did not undergo dehydration. If weigh-in had taken place right before competition, 89 % of the Elite (medal winners) would not have entered the weight category at which they competed. However, only 44 % of the non-Elite would not have entered the weight category they ended up competing in.

## Discussion

In this study we measured upper and lower body muscle power and isometric strength in 163 Olympic weight-class athletes (e.g. wrestling, boxing and taekwondo) at the official weigh-in (PRE) and 13–18 h after (POST), immediately prior to a National Championship competition. We found that based on urine osmolality, at weigh-in, 42 % of the athletes were moderately hypohydrated (700–1080 mOsm · kg H_2_O^−1^; HYP), 42 % were severely hypohydrated (1081–1500 mOsm · kg H_2_O^−1^; S-HYP) and only 16 % were not hypohydrated (<700 mOsm · kg H_2_O^−1^; EUH). In the hypohydrated groups (i.e., HYP and S-HYP) 13–18 h after weigh-in, body mass was recovered by 1.2 and 3.1 %, respectively. This gain in body mass in this relatively short period of time is attributable mostly to body water restoration. This fast rehydration did not increase isometric grip strength. However, lower body (i.e., CMJ) and upper body (i.e., bench press) muscle power increased in the group which was more hypohydrated (i.e., S-HYP) compared to the other groups. Thus, although severe dehydration permits fighters to enter a lower weight category, it reduces their neuromuscular performance. However, weight regain between weigh-in and the beginning of the competition (i.e., 13–18 h rehydration) offsets at least part of muscle function losses.

We observed that medal winners in the National Championship ranked among the more hypohydrated subjects based in urine osmolality at weigh-in (i.e., PRE). However, urine osmolality at weight regain (i.e., POST) was not different between the winners and the rest of the athletes (907 vs. 888 mOsm · kg H_2_O^−1^). This suggests that medal winners are able to dehydrate more than their counterparts and recover more fluid during those 13–18 h before competition. This allows them to be placed in a lower weight category at weigh-in, while relapsing to their habitual body mass in a few hours. This denotes a special ability in the high level competitors for losing and gaining body fluids. The relative change in bench press MPV in medal winners was larger than in the rest of the participants, which suggests that their ability to recover body fluids allows them to also rapidly recover their muscle power. Our data is in agreement with a previous study on 159 varsity wrestlers [8] reinforcing the belief that weight cutting and placement in a lower weight category could be associated with greater competition success.

Other authors have suggested the relationship between an athlete’s ability to weight-cut and their success in competition in combat sports. Horswill [2] pointed out that successful USA collegiate wrestlers at their national championship tended to lose more weight than those not as successful. Wroble and Moxley [8] suggested that wrestling below minimum wrestling weight is associated with greater success in competition. Lastly, Artioli’s group detected, in a descriptive study involving more than 800 judo athletes, that the level of aggressiveness in their weight loss behaviors (i.e., larger weight losses in shorter time) increases in the Elite [27]. However, our study is unique in explaining the association between weight loss at weigh-in and competitive success the next day. First, our measures of U_OSM_ allows us to confirm that in combat sport athletes, the loss of body mass involves dehydration, since the gains in body mass are tightly correlated with the reductions in U_OSM_ (*r* = 0.504; *p* < 0.001; Fig. 3), a recognized marker of hydration status. Secondly, our study suggests that muscle power in upper and lower muscles is reduced mostly when hypohydration is severe (i.e., S-HYP). Furthermore, our data shows that muscle power can be recovered (3-7 % in S-HYP) in the 13–18 h between weigh-in and competition (Figs. 4 and 5). We are not able to conclude if rehydration during those 13–18 h allowed full or partial muscle power restoration because we are lacking a basal measurement. However, the increases were higher in the elite group since they were more dehydrated at weigh-in. This suggests that although hydration and likely muscle power were not fully restored, entering a lower weight category compensated for those effects.

The group of athletes we tested included 39 women and 124 men of different competitive levels (National and International), with a wide range of body mass (45.8 to 119.8 kg), fat free mass (71.1 % to 97.7 %) and thus amount of muscle mass and potential strength. As a consequence, within each hydration level group these athletes present a disparity of neuromuscular performance in absolute terms for CMJ power, bench press MPV and isometric grip strength. For instance, a body mass gain between PRE and POST of 1000 gr in an individual of 50 kg (2 % increment) will expectedly have larger physiological consequences than the same rise in an individual of 100 kg (1 % increment). In the same way, an isometric grip strength gain of 2 kg between PRE and POST in an individual of 30 kg of maximum isometric grip strength (6.6 % increment) will be more significant than the same rise in an individual with a maximum grip strength of 60 kg (3.3 % increment). In contrast, a urine osmolality increment between the official weigh-in and the beginning of the tournament of 150 mOsm · kg H_2_O^−1^ has the same physiological effect, and therefore will have the same significance, in an individual of 50 kg, 70 kg or 90 kg of body mass. Thus, body mass, hand grip, CMJ power and bench press muscle contraction velocity changes between PRE and POST trials were analyzed in relative terms (i.e., percent changes), while urine osmolality values were analyzed in absolute terms.

The effects of hypohydration on muscle performance have been studied using different protocols and measurement techniques. Studies vary in the percentage of dehydration achieved from 1.7 % to 5.8 % of body mass reduction [13, 16, 20, 21, 28]. Also, in the mode of dehydration, with either thermally induced passive dehydration [29], exercise-induced active dehydration [20, 30] or diuretic induced dehydration [28]. Additionally, studies widely differ in the muscle performance tests used with studies testing either 1 RM [16], local muscle endurance [31], isometric strength [17, 20, 21, 31] or isokinetic force [19, 20]. Recently, Judelson and co-workers [32] reviewed the literature in this area and concluded that dehydration ranged between 2.5–5.0 % of body mass consistently attenuates strength by 2 % and power by approximately 3 %. The origin of these reductions has been speculated to reside on alterations in cardiovascular, metabolic or buffering functions [2]. Unfortunately, none of these factors have been able to be related to the losses in strength and power with hypohydration. Alternatively, hypohydration may affect neuromuscular function [20, 32] although membrane excitability is not reduced by dehydration [33]. In the present study we use a neuromuscular test in a real sports situation. Our subjects were left to dehydrate using a technique of their choice and their upper body neuromuscular performance was tested before and after rehydration using a very reliable technique [23]. We were able to discriminate a 1.5 % significant increases in mean propulsive velocity (MPV) due to the high reproducibility and sensitivity of this technique [34].

Our neuromuscular bench press testing is highly normalized and has high reproducibility [23] and sensibility [34–36]. However, neuromuscular function is influenced by circadian rhythm [34, 37]. We have reported 5.6–8.6 % reductions in bench press muscle power in the morning (8:00 h) in comparison to the afternoon (i.e., 18:00 h; [34, 36, 37]). Thus, the lack of increase in bench press muscle power in the group that recovered 1.2 % of their body mass (i.e., HYP) could be partially due to the fact that the test after rehydration (POST) was conducted in the morning (between 8:00 h and 10:00 h), while the hypohydrated test (PRE) was conducted in the evening (between 16:00 h and 19:00 h). Likewise, the percentage increases in neuromuscular performance found in S-HYP athletes (i.e., 2.8–7.3 %) could have been larger if they had been tested at the same time of day. Judging from urine osmolality, HYP and S-HYP subjects did not return to a euhydrated condition after the 18–24 h. However, S-HYP subjects significantly increased muscle power although they were still moderately hypohydrated (urine osmolality 1000.4 ± 23.0 mOsm · kg H_2_O^−1^; Fig. 1). It is possible that full rehydration would have resulted in larger gains in neuromuscular performance.

Grip strength was not sensitive to weight regain (rehydration) in our subjects. Isometric force has been previously evaluated in athletes prior and after dehydration but the results are controversial. Maximal isometric muscle strength is found to be either reduced [17] or unchanged [18, 19], probably due to the poor reliability of this measure (CV > 10 %). Furthermore, the effects of hypohydration on the central or peripheral nervous system is not evident when contraction time is not a limitation for motor unit recruitment, as it is during isometric contraction. Coinciding with our results, Judelson and co-workers, reported reduced central drive with hypohydration (reduced voluntary activation) however, isometric strength was unchanged [31].

Some authors sustain that the time allowed between weigh-in and competition is enough to recover fluid and energy substrates. Tarnopolsky et al. [38], observed that the weight loss using energy and fluid restriction before weigh-in results in a marked decrease in muscle glycogen concentration which could affect high intensity anaerobic actions common in combat sports. However, those reductions in muscle glycogen concentrations are largely reversed during the 17 h period allowed between weigh-in and the start of the competition [38]. Recent reports sustain that the dehydration incurred at weigh-in by combat sport athletes does not affect their combat performance. Artioli and co-workers studied judo combat athletes before and after rapid (5 to 7 days) weight loss (i.e., 5 %) using a self-selected regime that included voluntary dehydration. They found that weight loss did not affect judo-related performance (i.e., 5 min simulated combat) or anaerobic power during a Wingate test (i.e., 30 s all-out effort; [39]). Rapid weight loss did not affect performance in judo combat athletes that were not used to weight-cut (non-weight cyclers) and thus could not be attributable to adaptations to chronic weight cycling [40]. Thus, it is possible that the increase in muscle function that we found after 13–18 h of recovery in the S-HYP group which showed a decline in muscle power when hypohydrated at weigh-in (hypohydration) may not influence combat performance.

## Conclusions

In summary, our findings suggest that hypohydration is highly prevalent (i.e., 84 %) among competitive combat sports athletes, while 42 % of them undergo severe dehydration at weigh-in. Furthermore, severe dehydration at weigh-in (group S-HYP) seemed to lower neuromuscular bench press muscle contraction velocity (7.3 ± 2.6 %) and jumping power (2.8 ± 3.9 %) when compared to values after 13–18 h of rest and rehydration. Conversely, the neuromuscular performance impairment of severe dehydration can be reversed with rehydration during the few hours that elapse between weigh-in and competition. Our experimental design precludes us from ascertaining if this recovery of power is partial or full since we do not have a euhydrated control situation to compare it to. Finally, our data suggest that most successful competitors (medal winners in these National Championships) undergo severe dehydration followed by weight regain without reaching full rehydration. Perhaps, the advantage of competing in a weight category below the athlete’s habitual weight, balances the negative effects of competing somewhat hypohydrated.
